# OPREVENT2: Design of a multi-institutional intervention for obesity control and prevention for American Indian adults

**DOI:** 10.1186/s12889-017-4018-0

**Published:** 2017-01-23

**Authors:** Joel Gittelsohn, Brittany Jock, Leslie Redmond, Sheila Fleischhacker, Thomas Eckmann, Sara N. Bleich, Hong Loh, Elizabeth Ogburn, Preety Gadhoke, Jacqueline Swartz, Marla Pardilla, Benjamin Caballero

**Affiliations:** 1 Johns Hopkins University, Bloomberg School of Public Health, 615 N. Wolfe St. Suite W2041, Baltimore, MD 21205 USA; 2 National Institute of Diabetes, Digestive and Kidney Diseases Office of Nutrition Research, Bethesda, USA; 3 Harvard T.H. Chan School of Public Health, Boston, USA; 4 St. John’s University, Queens, NY USA

**Keywords:** Obesity, Adults, Rural, American Indian, Multi-level interventions, Policy, Study design

## Abstract

**Background:**

Obesity and other nutrition-related chronic disease rates are high in American Indian (AI) populations, and an urgent need exists to identify evidence-based strategies for prevention and treatment. Multi-level, multi-component (MLMC) interventions are needed, but there are significant knowledge gaps on how to deliver these types of interventions in low-income rural AI communities.

**Methods:**

OPREVENT2 is a MLMC intervention targeting AI adults living in six rural reservations in New Mexico and Wisconsin. Aiming to prevent and reduce obesity in adults by working at multiple levels of the food and physical activity (PA) environments, OPREVENT2 focuses on evidence-based strategies known to increase access to, demand for, and consumption of healthier foods and beverages, and increase worksite and home-based opportunities for PA. OPREVENT2 works to create systems-level change by partnering with tribal stakeholders, multiple levels of the food and PA environment (food stores, worksites, schools), and the social environment (children as change agents, families, social media). Extensive evaluation will be conducted at each level of the intervention to assess effectiveness via process and impact measures.

**Discussion:**

Novel aspects of OPREVENT2 include: active engagement with stakeholders at many levels (policy, institutional, and at multiple levels of the food and PA system); use of community-based strategies to engage policymakers and other key stakeholders (community workshops, action committees); emphasis on both the built environment (intervening with retail food sources) and the social environment. This paper describes the design of the intervention and the evaluation plan of the OPREVENT2.

**Trial registration:**

Clinical Trial Registration: NCT02803853 (June 10, 2016)

## Background

Obesity disproportionately impacts American Indian (AI) populations. The Centers for Disease Control estimated that roughly two thirds of AI adults are overweight or obese [1]. AI are more than 60% more likely to be obese than non-Hispanic whites [2], and have highest rate of type 2 diabetes of any ethnic population in the U.S. [3, 4]. AI adults are more than are twice as likely to be diagnosed with heart disease and coronary artery disease [5], 1.3 times more likely to have high blood pressure, and 2.4 times more likely to have a stroke [6].

There are multiple causes of higher chronic disease rates among AI communities. Most AI communities are rural, with a high proportion of households at or below the federal poverty level -- at the highest rate (28%) of any ethnic group in the U.S. [7]. Rural AI communities tend to have reduced access to paved roads, public transportation and retail food outlets, such as supermarkets. The retail food sources that are present in AI communities tend to carry a limited range of foods [8]. Many AI communitie﻿s are dependent on gas-station stores, which primarily stock energy dense, high fat, and high sodium items (e.g., sodas, chips, candy) and rarely carry fresh produce. Even when on-reservation stores stock healthier foods, they tend to be at a higher price than unhealthy items [9, 10]. Accessing affordable, high-quality healthy foods often requires off-reservation travel, and can easily be greater than 30 miles [8].

The built environment, including land use patterns, is also associated with low physical activity (PA) levels [11] and can be particularly influential in AI communities where resources allocated to the development of recreation facilities and parks are severely limited. Physical inactivity is more prevalent among AI adults as compared to non-Hispanic whites, and reports show that 72.8% of AI or Alaska Native adults aged 18 years or older do not meet federal PA guidelines [12]. Several studies have also found low PA and decreased leisure time activity in AI populations [13, 14].

Over the last decade, a number of studies have examined the development, implementation and evaluation of policy, systems and environmental changes at the local, state, and federal levels to promote active living and healthy eating [15, 16]. Unfortunately, few policy strategies have focused on AI communities [8, 17]. Many AIs live in separate reservation-based communities with tribal sovereignty and are relatively unaffected by mainstream obesity prevention policies. In areas where AIs are impacted by national or regional legislation, these policy approaches may not be sufficiently tailored to the resource constraints, cultural values or tribal sovereignty of the AI community [8]. As one example, the American Indian Healthy Eating (AIHE) Project was one of the first studies to systematically explore with tribal leaders their potential to utilize policy, systems and environmental change to promote healthy eating. Tribe-driven strategies were developed based on formative research that integrated qualitative, spatial, and policy analyses [8]. Building on the momentum established through AIHE, a capacity building project known as Healthy, Native North Carolinians (HNNC) was undertaken.

The majority of the obesity prevention trials conducted in AI communities has centered on children in the school setting [18–20]. Most of these earlier trials had modest success in changing behavior, with no impact on obesity [21, 22]. Despite the apparent success of these trials in changing the school food and PA environments, the community and household environments were unchanged, apparently reducing or even erasing overall health impact [21, 23, 24]. In the past decade, multi-level, multicomponent (MLMC) interventions have sought to intervene in multiple settings – and to change multiple aspects of the food and PA environments – in an effort to enhance potential to have health impacts. These studies include the successful Shape Up Somerville (SUS) and Baltimore Healthy Eating Zones (BHEZ) [25, 26] trials aimed at reducing childhood obesity.

To date, only one MLMC trial has centered on reducing and preventing adult obesity in AI communities. OPREVENT, the precursor to the current trial, was a pilot study that sought to reduce adult obesity in five AI communities. OPREVENT was a MLMC trial that worked in food stores, worksites, schools and through community media. The results of this trial were also modest, showing no impact on PA, and limited impacts on diet and psychosocial factors (unpublished data). No change in body mass inde﻿x (BMI) was found. Limitations of this previous work include: lack of attention to policy, which is needed for sustainability; lack of emphasis on PA; and weaknesses in the delivery of the intervention, such that intensity and exposure were limited.

**Fig. 1:**
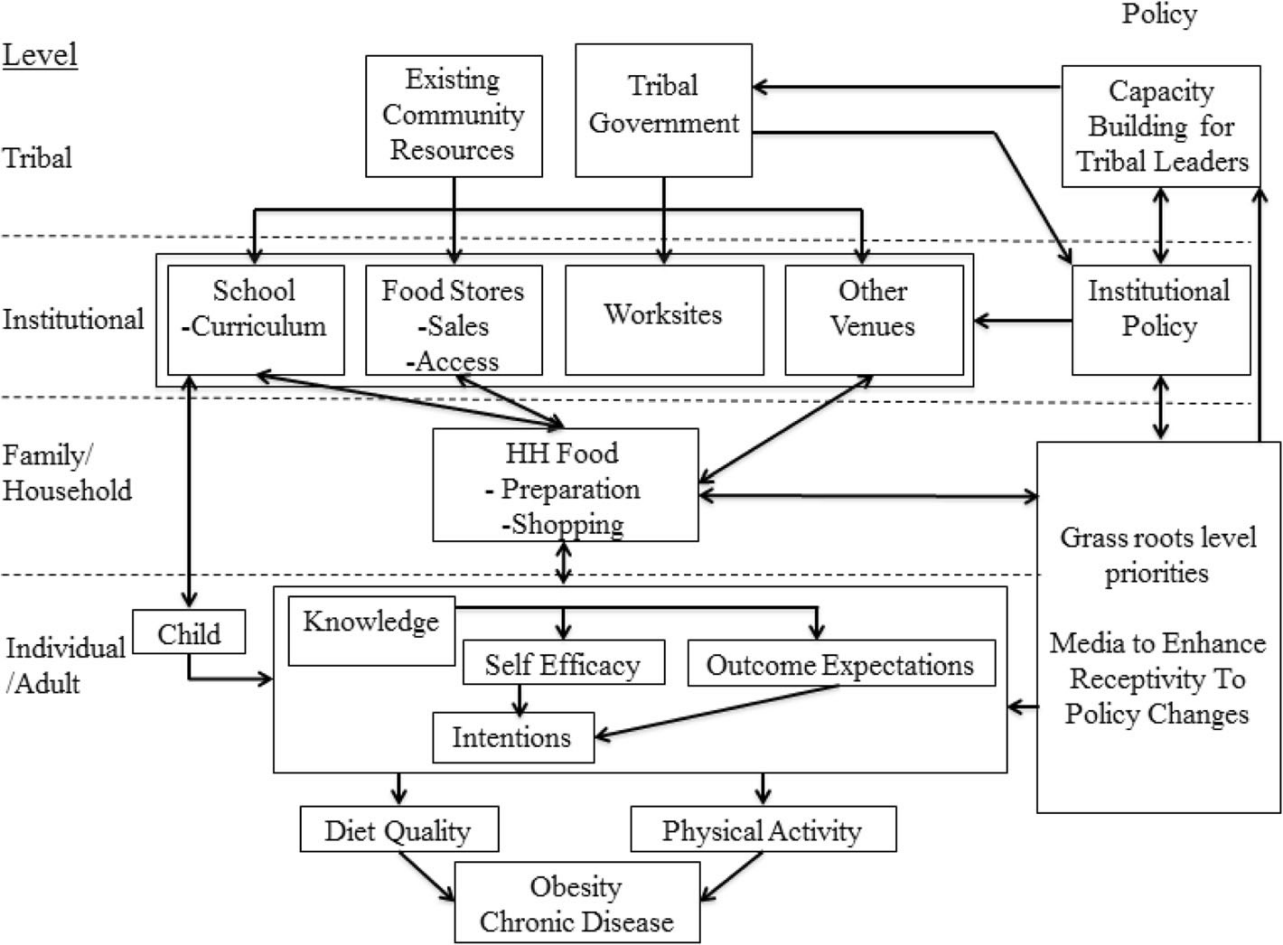
OPREVENT2 study conceptual framework

OPREVENT2 is a full-scale MLMC trial, aimed at adult obesity reduction and prevention in AI communities. The OPREVENT2 intervention is guided by Bandura’s social cognitive theory (SCT) and Bronfenbrenner’s social ecological model [27, 28]. SCT and the social ecological model conceptualize the individual as situated within broad family, institutional, community and political networks that influence their perceptions, behavior, and ultimately, their health status (Fig. 1). Psychosocial factors (e.g., knowledge, self-efficacy, behavioral intentions), social and interpersonal factors (e.g., family, peers), and physical-environmental factors (e.g., access to PA resources, food availability, price) interact at various levels to shape health outcomes. According to the social ecological model, these various levels and components operate as one interacting system within which information and resources flow bi-directionally from one level to another to influence health behaviors. Policy, institutional, and behavioral interventions have the potential to impact multiple levels of the food and PA environments.

OPREVENT2 seeks to develop and test a series of intervention strategies that will function at multiple levels. It will be implemented in collaboration with tribal policymakers, school administrators and teachers, worksites, and retail food stores. Institutional level intervention components will promote healthy food and PA-related behaviors in order to influence the household (e.g., food purchasing, preparation, sedentary behavior) and individual psychosocial factors and behaviors that will ultimately impact obesity.

## Methods/design

The OPREVENT2 trial uses a stratified, group randomized study design, where six AI communities have been selected to serve as either intervention (*n* = 3) or comparison (*n* = 3, delayed intervention) areas. Two communities are located in the Upper Midwest, and four communities in the U.S. Southwestern regions. Initially, 37 AI communities in both regions were contacted and invited to participate. The six participating communities are those who have agreed to participate and provided approvals in the form of tribal/chapter resolutions, as well as school and health board approvals. The research was approved by the Johns Hopkins Bloomberg School of Public Health Institutional Review Board (IRB) as well as the Indian Health Service IRB and the Navajo Nation Human Research Review Board. Written informed consent will be obtained for all participants in the impact evaluation.

### Formative research

Prior to the OPREVENT2 intervention in main trial communities, extensive formative research was conducted with two pilot AI communities: one in the Midwest and one in the Southwest region of the U.S. These communities are not OPREVENT2 main trial communities, though they have similar tribal affiliations and are based in similar regions as the main trial communities. The formative research focused on identifying how environmental changes are made throughout the communities. It also aimed to identify the facilitators and barriers these communities face in this process to identify ways to promote and support policy and environmental changes in the OPREVENT2 trial. In-depth interviews, observations, and modified talking circles were conducted in each pilot community [29]. Key participants included: tribal policymakers, health staff, and community member health champions. Theoretical and snowball sampling were used to identify additional participants engaged in the policy and structural change development processes in each community. Following and concurrent to this formative research, a small pilot was conducted in each community, consisting of three Community Action Committee meetings in each community. Participatory methods used in this pilot included talking circles, social mapping, and exploring environmental changes [30].

### Community workshops

The aim of the community workshops, which are currently underway, is to obtain in-depth input from the OPREVENT2 main trial community members regarding how to adapt previous OPREVENT intervention materials, and to define new materials/approaches that may be needed. Three different types of workshops are being conducted in each community: 1) community members focused on general intervention strategies and communications materials; 2) teachers and other school personnel focused on the school program component, and 3) tribal leaders and health staff that will inform structural and policy changes possible in each community. The research team will then review input and make final changes to the OPREVENT2 strategy and materials.

### Participants and recruitment

The target group in the OPREVENT2 MLMC intervention is AI adults, ages 18–75 years. Participants in the evaluation sample will be randomly recruited from household lists provided by each tribe/community. Due to privacy concerns, in four of the communities, local authorities have agreed to provide us with randomly selected households. Once consent is received, interviews will be conducted with one person per household until 80 have been completed for each of the six communities. Potential participants will be screened for eligibility (e.g., tribal member, adult 18–75 years, no plans to move for next two years). If a randomly selected household is unable to complete the interview, then the next eligible household will be selected from the recruitment list.

### Intervention

The OPREVENT2 intervention will involve multiple components at many levels (Fig. 1): policy, schools, fo﻿od stores, worksites, and community media (including social media). Each component of the OPREVENT2 program will reinforce several other components – either by improving access to or increasing demand for healthy foods and PA. The OPREVENT2 program will be implemented in six 2-month phases, with each phase focusing on different foods, activities, and promotions.

### Policy-level approaches

Community Action Committees (CAC) will be established within each community to identify and support structural and environmental changes, in partnership with community partners. Participation in the committee will be open to all community members, and recruitment will be done using mass media communication in each community. Health staff and tribal policymakers will also be invited to participate. The recruitment goal of this committee will be to bring together participants who are already engaged in making environmental changes in the community – whether formally through health programs and health policy or informally by the actions of existing champions in the community. Forming relationships between stakeholders, aligning existing health programs to support environmental changes, and developing health policies will help to increase sustainability of the OPREVENT2 intervention and promote long-term environmental changes in each community. This committee will meet monthly, and will work to support: sustainability of the OPREVENT2 program and critical reflection of the community environment and its role in shaping behavior, and community member interest in the health fields. These meetings will be facilitated by an OPREVENT2 interventionist, who will also work to train a new facilitator in the community to enhance sustainability.

### Changing food access in community food stores

Our work with food sources will aim to increase access to healthier foods by working in all on-reservation stores that agree to participate. A central strategy will be increasing the stocking and sales of affordable healthy food options at food stores. For smaller, privately owned stores, we will provide gift cards to their wholesaler, enabling them to purchase a starter stock of promoted foods and beverages. We will conduct promotional activities at the point of purchase, including shelf labels, posters, interactive sessions such as taste tests and cooking demonstrations, and the provision of flyers, booklets, small gifts and other promotional materials. Interactive sessions will occur at least once per week in each participating food store. Our intervention components at the food source level are based on previous small store interventions conducted in AI communities [31, 32] with additional innovative pieces, including training videos to assist store owners/managers in stocking/marketing healthier foods.

### Increasing physical activity opportunities in worksites

Physical activity will be a primary focus of the OPREVENT2 intervention worksite component. Intervention materials for worksites will focus on educating community members on recommended type and duration of PA, making a plan and setting goals to exercise, including working out with a partner [33–35], and fueling PA with proper nutrition. Educational materials, including posters, flyers, and educational displays, will be made available at worksites. Interventionists will make several visits to worksites to present on the health benefits of PA.

The primary activity to promote PA in worksites will be a FitBit Challenge. Employees within each worksite will be encouraged to register in teams for the challenge. Interventionists will distribute free FitBits to registered teams and provide teams with an orientation on proper use, including how to track and monitor their participation in the challenge. Interventionists will make weekly follow-up visits to each worksite to record daily and weekly steps for challenge participants. The members of the team with the highest overall number of steps will be awarded each month with “Team of the Month” certificates. At the end of the Challenge, the final winning team will receive a free healthy lunch provided by OPREVENT2. The top three walkers from each OPREVENT2 community will be awarded a commemorative plaque.

### School curriculum, developing Native youth as household obesity change agents

A school-to-family curriculum aimed at encouraging children to be household change agents will be a critical component of the OPREVENT2 project. Children influence adults in their households positively for healthier nutrition and PA related habits on multiple levels [36–41]. School-based curricula have reported improved adult household members’ food getting habits, reduced fat intake and consumption, increased fruit and vegetable consumption, and lower BMI. In our precursor study, OPREVENT, we developed a unique, culturally relevant and sensitive school-based curriculum with the partnership of AI curriculum developers and artists for grades 2–6. The grades 2–4 curricula are comprised of weekly in-class curriculum materials that are tailored to each state’s education standards and an AI storybook. The storybook highlights a poignant story of two young children who learn about healthy lifestyles from their cousin, the change agent in their life, and develops into change agents for their household. The children travel and experience other tribal cultures, challenges, and strengths of each of the six AI communities participating in OPREVENT2. The grades 5–6 curriculum will adapt the “Cooking with Kids” tasting program [42]. Teachers in each school will be trained in the delivery of the curricula for their grade level, and will be provided with materials needed to implement the program in their classrooms.

### Community and social media

Community and social media will be an integral component of the OPREVENT2 intervention. Community media will be based on OPREVENT, which included use of newsletters and radio announcements within communities to spread intervention messages. Newsletters and radio announcements will be specific to each phase, and produced in the local Native language and delivered by Elders whenever possible. In addition to promotion of intervention messages, we will work with tribal health staff to implement community-wide activities, including walking clubs and food store tours. OPREVENT2 staff will attend existing community events such as health fairs, community meetings, school activities, and community walks to promote OPREVENT2 activities and to identify key community stakeholders for partnership. Study communities have a variety of local media, including local radio and cable access channels, bulletin boards, newsletters, and websites. Announcements through local media will be used to promote ongoing activities and products at schools and stores.

Social media and online networks have the capacity to disseminate information widely while promoting social support and reshaping norms [43, 44]. The OPREVENT2 social media component will consist of Facebook, Instagram, and Twitter platforms. A Social Media Working Group has been formed to explore the best message content for each platform. Facebook and Instagram messages will be focused on health information and current events pertinent to our population, such as common health issues and promotional activities in AI communities. Topics will range from exercises tips to healthy recipes incorporating traditional foods. The primary focus of Twitter will be to share recent research related to fitness and nutrition with other community stakeholders as well as with Native tribes/communities. Content published on each platform will be promoted and boosted using tools built into each platform for this purpose (e.g., boosting of Facebook posts, creation of Twitter campaigns) in an effort to increase followers and expand the reach of the intervention messages.

### Standards for intervention delivery, process evaluation

Intervention implementation at each level will be monitored through ongoing process evaluation, with the intention of assuring that set standards are being met (Table 1). These standards are based on our review of the literature, and on our previous experiences implementing MLMC interventions in AI communities. The OPREVENT2 intervention team will meet every two months to review the degree to which intervention standards are being met, and will improve implementation on that basis.

**Table 1:** Select process evaluation measures and standards by OPREVENT2 intervention component

Intervention level	Process evaluation measure	High standard^a^
Policy	# CAC meetings held/community/year # attendees/CAC meeting # different sectors represented/meeting	12 10 4
Schools	% teachers (grades 2–6) trained/school % lessons taught/grade % family packs returned	90 75 60
Food Stores	% promoted healthy foods stocked % shelf labels correctly placed # interactive sessions/store/phase # visitors/interactive session	80 80 6 20
Worksites	# participating worksites/community # coffee/water stations made over/participating community % AI staff initially participating in FitBit Challenge % AI staff participating in FitBit Challenge after 2 months	5 5 70 50
Community media	# radio announcements/week # newsletter/mailed flyers/phase	3 4
Social media	# Facebook, Instagram, Twitter posts/day/platform # boosts Facebook, Instagram, Twitter/week/platform # likes/follows/week/platform	3 3 50

^a^Quality of implementation based on % high standard met: low = <50%, med = 50–99%, high= > =100%

### Comparison communities (delayed intervention)

The comparison communities will receive the OPREVENT2 intervention following completion of the post-intervention evaluations.

### Data safety and monitoring

A Data Safety and Monitoring Board, meeting 1-2 times per year, has been formed to assure the study meets standards of safety and confidentiality, and to deal with possible adverse events.

### Measurements

The OPREVENT2 trial will be evaluated at each intervention level (Table 2). Process evaluation measures will assess reach, dose delivered, and fidelity of intervention implementation (Table 1).

**Table 2:** Impact evaluation measures for the OPREVENT2 trial by intervention level

Intervention level	Impact/outcome measure
Policy	# CAC action items achieved/year # health-related issues put on tribal policymaker’s agenda/year Increase in CAC participant collective efficacy scores
Schools	# policy changes made to improve the school food and PA environments # structural changes made to improve the school food and PA environments (e.g., removal of vending machines)
Food Stores	# units of promoted foods sold # units of promoted foods purchased/consumed by OPREVENT2 participants
Worksites	# policy changes made to improve the workplace food and PA environments # structural changes made to improve the workplace food and PA environments (e.g., changing what’s in vending machines)
Adult/household	Household food purchasing (healthy and unhealthy foods) Healthiness of common methods of food preparation Change in psychosocial factors (knowledge, self-efficacy, intentions) Change in dietary patterns (e.g., total calories, total fat, FV servings, HEI scores, etc.) Change in PA (IPAQ, FitBit) Change in weight, BMI, blood pressure, waist and hip circumference

A sample of adults (100 per community) will be surveyed pre and post intervention to assess impact.

The Adult Impact Questionnaire (AIQ) will assess the food getting frequency of our promoted products, household patterns of food acquisition and preparation, adult PA, morbidity history and sociodemographic variables (e.g., age, education, income range). In addition, the AIQ will evaluate psychosocial constructs including healthy food knowledge, self-efficacy, intentions about food, and community resources/environment. The AIQ instrument uses a modified Individual Physical Activity Questionnaire (IPAQ) Short Form (IPAQ-SF). The IPAQ-SF specifically asks about walking, moderate-intensity PA, and vigorous-intensity PA. It was determined to be appropriate for this population and study for several reasons: validity studies have been conducted in similar populations; low time burden it was easy to modify to be more culturally acceptable for our target population; and it was piloted in the communities and found to be acceptable. Sociodemographic, family medical history and anthropometric measurements (i.e., height, weight, percent body fat, waist circumference, hip circumference, blood pressure, heart rate) will be collected to assess obesity and other health-related factors. We will be using Tanita 300GS (Tanita Corp., Tokyo, Japan) scales for body weight and percent body fat, and the Omron Fat Loss Monitor (HBF-306) will be used for a second percent body fat estimate. Blood pressure and heart rate will be measured using an Omron Automatic Wrist Blood Pressure Monitor, Model #BP652 (HEM-6052-Z), and will be assessed pre and post intervention in intervention and comparison respondents.

We worked with NutritionQuest to develop a modified Block Food Frequency Questionnaire (FFQ) specific to our study sample. In addition to standard Block FFQ food items, it includes traditional AI foods common in the Southwest and Upper Midwest regions, as well as specific food items promoted and de-promoted within our intervention. The FFQ was based on the FFQ that was used in the Strong Heart Study Family Study, Cardiovascular Disease in American Indians (Phase V), an instrument used by investigators at the Johns Hopkins Center for American Indian Health. The FFQ includes 95 questions pertaining to frequency and portion size of particular foods. Portion sizes will be estimated with the aid of standard portion size illustrations. The final section of the FFQ includes 24 questions pertaining to additions to food items (e.g., milk, creamer, etc. to coffee) and type of food items (e.g., low-fat, low-carb, etc.). The FFQ will be used to assess dietary intake pre and post intervention in intervention and comparison respondents.

At post intervention data collection, we will collect information on exposure to specific intervention components and materials for all participants which will be used to assess self-reported dose received by our evaluation sample.

A series of structured surveys will be conducted pre and post intervention in schools, food stores, and worksites to assess change at the level of these institutions in terms of the food and PA environments. A Community Action Component Impact Questionnaire will be administered to committee participants at their first meeting and at post intervention. Participant characteristics (e.g., age, occupation) will be gathered, and the instrument will be used to gauge change in collective efficacy, intentions, as well as a free list of community changes and feedback of CAC activities (post intervention only).

### Data safety and confidentiality

Electronically collected data will be encrypted using Secure Sockets Layer (SSL) encryption using REDCap and will be upload to a server at Johns Hopkins Bloomberg School of Public Health. All data collectors received a unique user PIN that are ciphered using Secure Hash Algorithm (SHA) cryptography, and all stored REDCAP data and REDCAP application logs are encrypted using advanced encryption standards on tablet PC until the data is uploaded. A separate administrator username and password will prevent data collectors from altering the data and data collection instruments.

### Sample size and statistical methods

Sample size calculations were based on testing the change in mean BMI. Modeling and calculations were based on testing for an effect size d = |μ1–μ2|/σ = 0.5, where μ1 is the mean change in one of the two primary outcome measurements in the treatment group, μ2 is the corresponding mean change in the control group, and σ is the standard deviation of the observed differences. The detectable difference (|μ1–μ2|) for the change in mean BMI and daily FV intake was determined using the impact data from OPREVENT and other interventions similar to OPREVENT2. We used OPREVENT baseline BMI data on 422 AI adults to estimate the standard deviation of the observed differences between pre and post intervention BMIs, assuming that the variances of mean BMI from pre and post intervention are equal and assuming a large correlation between pre- and post-intervention measurements (*r* = 0.9). We used data from a survey sample of AIs in the Midwest [45] to estimate the standard deviation of the observed differences in mean daily FV under the same assumptions. For BMI, σ = 3.5 and therefore the detectable change in average BMI is 1.3 kg/m^2^. For FV, σ = 1.56 and therefore the detectable change in daily FV servings is 0.78 servings.

The power was obtained from a two-sample t-test formula β = 1 ‐ P(t ≤ t_α/2_) + P(t  ≤ ‐ t_α/2_), where t is a non-central t-distribution with df = 2(3–1) degrees of freedom and a non-centrality parameter NC = d/[2{1 + (85–1)ρ}/(3*85)]^1/2^. Here ρ is the within-community correlation between the differences between the primary outcomes. Calculations were based on a ρ equal to 0.01; this value is consistent with the range of correlations reported in the literature [46, 47] and the correlation for baseline BMI and FV found in OPREVENT. All calculations were done assuming a 20% loss to follow-up from pre to post data collection.

With 80 participants per community and three communities per arm, there is 80% power to detect an effect size of d = 0.5 (which corresponds to a 1.3 kg/m2 change in mean BMI and a 0.78 change in mean daily fruit and vegetable consumption) with a type I error of 5%.

We will examine intervention effects by subtracting the pre intervention score/intake from post intervention score/intake and then conduct linear mixed-effect models for confirmatory testing of intervention effects. We will conduct t-tests for normally distributed continuous variables and nonparametric Wilcoxon-Mann-Whitney tests for non-normal continuous variables; Chi square and Fisher’s exact tests for dichotomous variables; and Mantel trend tests for ordinal variables. We will follow this exploratory analysis with multilevel mixed models using the lmer function from the lme4 package for R [48]. Linear and logistic regression models will be adopted for identifying intervention effects as well as risk factors in regression analyses. We will examine multiple primary response variables, including specific indicators of dietary quality (e.g., FV servings, energy intake) and of obesity (e.g. BMI). Fixed effects of the model will be baseline values for these variables, treatment group, age, gender, ethnicity and mediating variables wherever applicable. The community will be treated as a random effect.

## Discussion

To our knowledge, OPREVENT2 will be one of only two MLMC obesity prevention intervention trials targeting AI adults. Substantial formative research and a community engagement process will be used to refine and develop intervention strategies and materials. OPREVENT2 will be a unique trial that integrates stakeholders at multiple critical levels: policy, food retail, worksites, schools, and individual. Multiple components of the food and PA environments will be targeted, leading to improved exposure and reinforcement of key messages. There will be an emphasis on social dimensions of the environment, involving social media and the use of children as change agents in the home. Detailed process and impact evaluations will occur at all intervention levels.

Tribal leaders, tribal health staff, researchers, and public health practitioners will be greatly interested in OPREVENT2 program findings. MLMC interventions are thought to be required to address the multifactorial causes of obesity, yet few of these large intervention trials have been successfully completed. Trials such as OPREVENT2 are required to see if MLMC approaches are indeed more successful, and how they should best be implemented.

One major strength of the OPREVENT2 program will be that it will provide a model of how to work with tribal policymakers and other key stakeholders to improve the community food and PA environments. Because of its prolonged and frequent engagement with policymakers and other community leaders, OPREVENT2 may lead to long-term impact, and be sustained through institutionalization of intervention components.
